# Inflammation, Dopaminergic Brain and Bilirubin

**DOI:** 10.3390/ijms241411478

**Published:** 2023-07-14

**Authors:** Sri Jayanti, Camilla Dalla Verde, Claudio Tiribelli, Silvia Gazzin

**Affiliations:** 1 Italian Liver Foundation, Liver Brain Unit “Rita Moretti”, Area Science Park, Bldg. Q, SS 14, Km 163,5, 34149 Trieste, Italy; sri.jayanti@fegato.it (S.J.); camilla.dallaverde@fegato.it (C.D.V.); silvia.gazzin@fegato.it (S.G.); 2 Eijkman Research Centre for Molecular Biology, Research Organization for Health, National Research and Innovation Agency, Cibinong 16915, Indonesia

**Keywords:** addiction, cognition, depression, schizophrenia, Parkinson’s disease, attention deficit hyperactivity disorder, tumor necrosis factor, PPARs, NFkβ, MAPKs

## Abstract

Dopamine is a well-known neurotransmitter due to its involvement in Parkinson’s disease (PD). Dopamine is not only involved in PD but also controls multiple mental and physical activities, such as the pleasure of food, friends and loved ones, music, art, mood, cognition, motivation, fear, affective disorders, addiction, attention deficit disorder, depression, and schizophrenia. Dopaminergic neurons (DOPAn) are susceptible to stressors, and inflammation is a recognized risk for neuronal malfunctioning and cell death in major neurodegenerative diseases. Less is known for non-neurodegenerative conditions. Among the endogenous defenses, bilirubin, a heme metabolite, has been shown to possess important anti-inflammatory activity and, most importantly, to prevent DOPAn demise in an ex vivo model of PD by acting on the tumor necrosis factor-alpha (TNFα). This review summarizes the evidence linking DOPAn, inflammation (when possible, specifically TNFα), and bilirubin as an anti-inflammatory in order to understand what is known, the gaps that need filling, and the hypotheses of anti-inflammatory strategies to preserve dopamine homeostasis with bilirubin included.

## 1. Introduction

The impairment of the physiologic dopamine (DA) function and the death of dopaminergic neurons (DOPAns) bring Parkinson’s disease immediately to mind; however, DA affects extensive tasks in the brain. DA is involved in pleasure, sleep, mood, cognition, motivational salience, reward-motivated behavior, hormone release, addiction, depression, schizophrenia, attention deficit hyperactivity disorder, multiple sclerosis, ischemic stroke, and more [1,2,3,4,5,6]. Thus, the homeostasis of DA systems exceeds what would generally be perceived, involving both mental and physical health.

Like adrenaline and noradrenaline, DA is a catecholamine that possesses the 3,4-dihydroxy aromatic ring (the catechol core) synthesized from L-tyrosine, which could be detected in the whole body. The DA produced in the brain cannot pass the blood–brain barrier (BBB); however, peripheral organs can produce it and react with it thanks to the presence of five different receptors, which, in turn, act on multiple signaling pathways (see later). The DA cascade might also finally play a critical role in mammals’ whole body physiology, health, and diseases [7]. For example, DA controls the pathophysiology of the heart and cardiovascular system, gastrointestinal system, liver, innate and adaptive immunity, the kidney (see also Section 4), and the brain [7,8,9,10,11].

**Figure 1:**
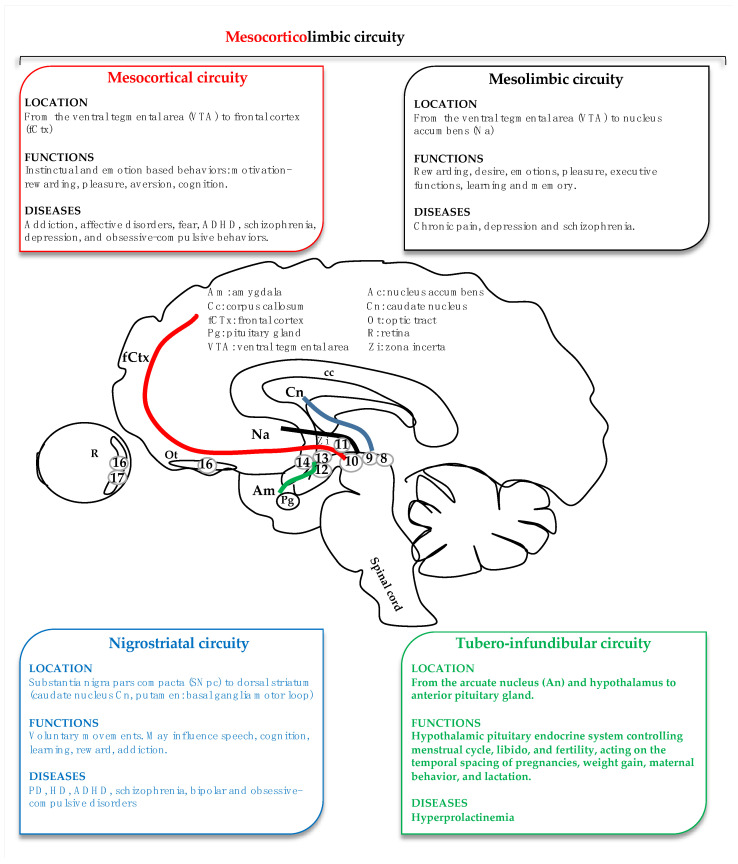
Cartoon of the four dopaminergic circuits, their major function, and the pathologic conditions affecting each circuit. Red: the mesocortical circuit; black: mesolimbic circuit; blue: nigrostriatal circuit; and green: tubero-infundibular circuit. Am: amygdala; Ac: nucleus accumbens; Cc: corpus callosum; Cn: caudate nucleus; fCTx: frontal cortex; Ot: optic tract; Pg: pituitary gland; R: retina; VTA: ventral tegmental area; Zi: zona incerta. Circles with numbers at the origin of each circuity indicate the histologic classification of the dopaminergic neurons. PD: Parkinson’s disease, HD: Huntington’s disease, ADHD: attention deficit hyperactivity disorder.

In the brain, DA is synthesized through a restricted population of cells (less than 1% of the total number of neurons in the central nervous system (CNS) that are located in specific areas [1,12,13] and form four major dopaminergic circuits (see later and Figure 1)). Of importance, DA could regulate both innate and adaptive immunity due to its ability to bind to cell receptors of both immune and neuronal systems as well those reviewed by Carandini et al. [14] and Mingan Li [11]. On the other hand, systemic inflammation has been reported to affect a broad range of neurologic and neurodegenerative conditions, with aging leading to a 4–7% DOPAn loss per decade and environment and lifestyle affecting the systemic inflammatory status and amplifying the mechanisms [10,11,14,15,16,17,18,19,20,21,22].

**Figure 2:**
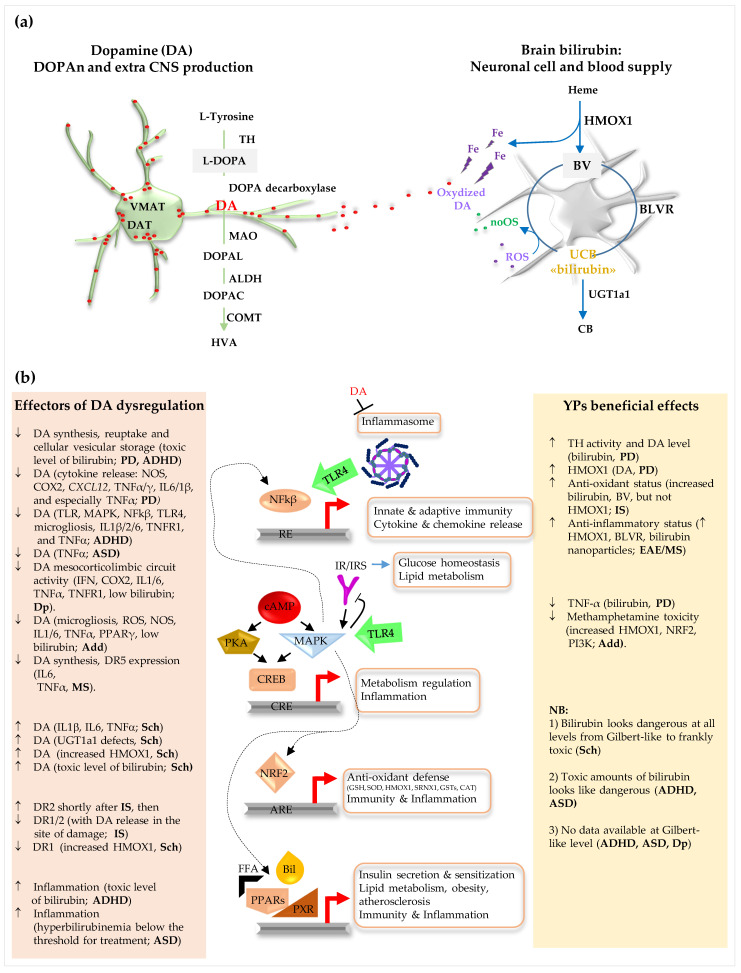
Dopamine and bilirubin metabolism, biological effects, and shared signaling pathway. (**a**) Cartoon showing dopamine (**left**) and bilirubin (**right**) metabolism. (**b**) Cartoon resuming the principal effectors of DA dysregulation in neuropsychiatric and neurodegenerative conditions described in this paper (left), and the potential beneficial effects of YPS based both on clinic and experimental information (right), and the main signaling pathway shared between the neurologic conditions and YPs (middle). ↑: increase; ↓: decrease; TH: tyrosine hydroxylase; L-DOPA: L-dihydroxyphenylalanine; DA: dopamine; MAO: monoamine oxidase; DOPAL: 3,4-dihydroxyphenylacetaldehyde; ALDH: aldehyde dehydrogenases; DOPAC: 3,4-dihydroxyphenylacetic acid; COMT: catechol-o-methyltransferase; HVA: homovanilic acid. Heme: hemoglobin; HMOX: heme oxygenase; BV: biliverdin; BLVR: biliverdin reductase; UCB: unconjugated bilirubin («bilirubin»); UGT1a1: uridine-glucuronosyl transferase 1a1; CB: conjugated bilirubin; ROS: reactive oxygen species; noOS: unreactive oxygen species; PD: Parkinson’s disease; NOS: nitrogen reactive species; COX2: cyclooxygenase 2; CXCL12: C-X-C motif chemokine ligand 12; TNF: tumor necrosis factor; IL: interleukin; TLR4: *Toll-Like Receptor 4*; MAPK: mitogen-activated protein kinase; NFkβ: Nuclear factor NF-kappa-B; RE: responsive elements; TNF-R1: tumor necrosis factor receptor 1; ADHD: attention deficit hyperactivity disorder; ASD: autism spectrum disorder; IFN: interferon; Dp: depression; PPAR: peroxisome proliferator-activated receptors; Add: addiction; DR5: dopamine receptor 5; MS: multiple sclerosis; Sch: schizophrenia; EAE: experimental autoimmune encephalomyelitis (model of MS. multiple sclerosis).

The brain and the body have increasingly been suggested as a single entity in terms of inflammation. Neuroinflammation is considered a driving mechanism and a possible marker in the occurrence, diagnosis, and treatment of neurodegenerative diseases and neurologic conditions [23]. In the case of DA, studies have consistently reported evidence of a correlation between increased chronic low-grade inflammation, elevated peripheral and central inflammatory cytokines, inflammatory mediators, and acute-phase reactants (interleukin: IL6, IL1; tumor necrosis factor: TNFα; C-reactive protein—CRP, etc.), with decreased DA availability as a subject with neuropsychiatric disorders [14,22,24]. Indeed, infectious diseases (HIV, sepsis), trauma, and TNFα administration as therapeutics in cancer have also been reported to induce depressive symptoms, with reduced motivation, anhedonia, and altered sleep [24]. Signaling pathways connecting inflammation and DOPAn degeneration have been suggested and evaluated as potential therapeutic targets. Among the most reported include cAMP/PKA/MAPK (cyclic adenosine monophosphate/protein kinase A/mitogen-activated protein kinases), NRF2 (nuclear factor erythroid 2–related factor 2), PPARs (peroxisome proliferator-activated receptors), NFκB (nuclear factor kappa-light-chain-enhancer of activated B cells), TLR4 (toll-like receptor), and inflammasome-NLRP3 (NOD-, LRR- and pyrin domain-containing protein 3) [4,10,11,15,18,22,25,26,27,28] (Figure 2).

Bilirubin is another body metabolite with pleiotropic and surprising functions, both at systemic and CNS levels. Mainly known as a waste product of hemoglobin catabolism possibly induces neuronal damage in infants and as a marker of liver dysfunction over the last decades, bilirubin has been rediscovered as a homeostatic, anti-oxidant, anti-inflammatory factor, and a hormone [29,30,31,32,33,34,35]. Epidemiologic studies have repeatedly reported an inverse correlation (protective) between mildly elevated total serum bilirubin (TSB) and chronic inflammatory conditions (e.g., diabetes, metabolic syndrome, cardiovascular diseases). At the same time, a low TSB (about or below the lower physiologic range of 0.4–1 mg/dL) has been associated with neurological conditions, suggesting that this pigment is a risk factor or a marker of CNS diseases [30,31,35]. The therapeutic application of bilirubin has started to be discussed [29,36,37]. Understanding the possible biomolecular effects on different neurologic diseases is mandatory from this perspective. Bilirubin is produced by the action of two sequential reactions. The first one is the conversion of hemoglobin to biliverdin, which is played by heme oxygenase (HMOX1); subsequently, biliverdin (BV) is converted to unconjugated bilirubin (UCB, or indirect bilirubin, simply bilirubin further in the review) by biliverdin reductase (BLVR) (Figure 2). Bilirubin, BV, HMOX1, and BLVR (the yellow players -YPs) activity and modulation in the course of this disease have been documented in the brain [35,38,39,40,41,42,43]. This high capability to modulate their level and activity, joined with documented benefits, has supported the idea that YPs are a form of homeostatic and defensive machinery in the cell. Moreover, YPs can act directly and indirectly (through signaling pathways, including—PPAR; MAPK; NRF2; AhR—aryl hydrocarbon receptor; NFkB; insulin pathway IRs/IRK/PI3K—insulin receptor substrates/insulin transmembrane receptor kinase/phosphatidylinositol 3-kinase [30,31,35]) on inflammation (e.g., TNFα, IL6, complement, T cell response, preventing the alteration of the BBB [44]). On the other hand, a sustained pro-inflammatory status usually down-regulates the YPs [44]. YPs are primarily known to modulate the cellular redox balance: another mechanism that is often reported in neurologic disease. According to the available data, this review aims to discuss the evidence connecting DOPAn, inflammation, and bilirubin.

## 2. The Dopamine Circuits in Brain

An in-depth review of the dopamine pathway is out of the scope of this review. The following section aims to provide a laid background on the DA pathway and DOPAn location in the brain to help readers visualize the complexity of dopamine’s function in human behavior and related neurologic conditions.

To accomplish this objective, we performed a search of the literature on PubMed, Scopus, and Google Scholar are as follows. (1) First, we searched for the most focused and relevant works in the literature using the keywords “dopamine”, or “dopamine system”, or “dopamine diseases/pathology and inflammation”. (2) Second, we searched for “dopamine and bilirubin, or HMOX, or BLVR, or BV”. To this step, we added the literature that was already part of our background on YPs. (3) Finally, after reading the most relevant publications, we refined the literature search using keywords that were the most promising specific terms to emerge from steps 1 and 2.

DOPAn are a heterogenic family of nine different subtypes of cells (A8–14, A16, A17), which are grouped in well-defined brain regions connecting topographically organized anatomical tracts [1,3,12] that are mostly localized in mesencephalon and diencephalon (Figure 1). They all express genes that are critical for dopamine catabolism (Figure 2) and survival [6,12,45].

DA is involved in four major circuits in the central nervous system: (a) The nigro-striatal circuity; (b) The tuberoinfundibular circuity; (c) The mesocortical circuity; and (d) the mesolimbic circuity, with the last two largely overlapped, thus possibly referring together to mesocorticolimbic circuity [46]. Altogether the four DA circuits described accounted for 90% of DOPAn in the brain [1]. Additional DOPAn were located in the retina, olfactory tubercle, and zona incerta [12] (Figure 1). The biology of DA in inflammatory conditions might be impressively variable, complicating the understanding of therapeutic approaches. These effects could depend on DA synthesis, trafficking, sequestration into vesicles, and release, from the type of DA receptors expressed in target tissues and cells (DA receptors: DR, G-coupled receptors type; with DR2, DR3, and DR4 usually inhibitory; and DR1, DR5 usually excitatory) from the concentration of the cytokine, the duration of the stimuli and even by the DA circuit involved [4,11,19,22,47]. Finally, the dysregulation of DA activity resulted in pathophysiology [13,26,48,49,50,51].

### 2.1. The Nigrostriatal Circuity

The nigrostriatal circuit originates from a small group of DOPAn (histologic identification A8, 9; 3–5% of total neurons of the area) located in the substantia nigra pars compacta (SNpc) and projects to the basal ganglia (dorsal striatum: caudate nucleus and putamen). This pathway mainly regulates voluntary body movement but has also been involved in procedural and associative learning, including speech, cognition, reward, and addiction. The neuropsychiatric and neurodegenerative conditions, usually referred to as the nigrostriatal circuit, accounted for HD, attention-deficit hyperactivity disorder, schizophrenia, bipolar disorders, and obviously, Parkinson’s disease (PD) [12,13,50,52,53,54,55,56,57,58,59,60].

### 2.2. The Mesolimbic Circuity

The DOPAn populating the ventral tegmental area (VTA, A10, 60–65% of the neurons of this region) projects to the ventral striatum (nucleus accumbens—Na, olfactory tubercle—Ot), the amygdala—Am, the hippocampus, and septum. This circuit is mainly involved in all instinctual and emotion-based behaviors (Figure 1), such as motivation, aversion, incentive salience, pleasure, reinforcements, reward, the determination of personality traits, and cognition. The dysregulation of this mesolimbic circuit underlies a whole plethora of neuropsychiatric diseases, including among them addiction, attention-deficit hyperactivity disorder, schizophrenia, depression, and obsessive-compulsive behaviors, as well as chronic pain. If usually the perturbation of the mesolimbic DA reward system has been linked to addiction to drugs, the correct meaning of the term is worth mentioning in that the same pathway could create a good lifestyle, such as “addiction” to sports, good food, responsible behavior for drugs, alcohol, etc. It may be of high relevance in adolescents [12,13,19,48,61,62,63,64].

### 2.3. The Mesocortical Circuity

The DOPAn of the VTA (A10) also projects to the prefrontal cerebral cortex, the cingulate cortex, and the perirhinal cortex (Figure 1). This circuit is implicated in cognition, executive functions, emotions, learning and memory, pleasure, and chronic pain. Depression and schizophrenia are frequently related to the malfunctioning of this DA circuit [12,13,62,63,64,65,66,67,68].

### 2.4. The Tuberoinfundibular Circuity

In the tuberoinfundibular circuit, DOPAn secretes DA from the arcuate (A12) and periventricular nuclei of the hypothalamus (A14) to the anterior pituitary gland. This pathway controls the hypothalamic-pituitary endocrine system and inhibits prolactin secretion from the anterior pituitary gland into the blood, mainly affecting fertility and maternal behavior. As a matter of notice, treatment with anti-dopaminergic anti-psychotic drugs (as an example in schizophrenia) causes hyperprolactinemia (Figure 1) [12,13,50,69,70,71].

These DA circuits are interrelated, which could explain the presence of mixed cognitive, executive, behavioral, psychotic, and motor abnormalities in PD and other conditions involving dopamine [72].

## 3. Neurological Conditions Related to Dopamine

### 3.1. Parkinson’s Disease

Parkinson’s disease (PD) is the most known neurodegenerative condition and is characterized by a progressive reduction in DA and DOPAn, with the number of people affected predicted to double from 6.9 million in 2015 to 14.2 million in 2040 [73]. The loss of DA in SNpc is responsible for motor disabilities (mainly: bradykinesia, tremors, dystonia, gait complaints, and falls). Other non-motor symptoms (e.g., olfactory impairment and constipation, apathy, sleep abnormalities, anxiety, depression, together with cognition problems) are attributable to alterations in the mesocorticolimbic system that usually precede the motor symptoms [5,21,53,74,75]. The etiology of PD has not been fully elucidated. In 90% of cases, no genetic identifiable explanation can be obtained. Multiple possible triggers have been suggested, as reviewed by several authors. Among these triggers, environmental factors (metals, pesticides, and herbicides), lifestyle (especially the high consumption of milk and dairy products [76,77]), gut dysbiosis (by both the induction of a systemic pro-inflammatory status as well as by direct signaling through the vagus [21,78]), abuse substances (e.g., methamphetamine—by impairing DA transport and increasing its extracellular concentration), the metabolic syndrome (namely insulin resistance through PPARs signaling), brain trauma (by breaking the BBB and inducing inflammation), and physiologic aging, accompanied by the so-called “senescence neuroinflammation”, contribute or even are supposed to trigger DA loss [10,15,76,78].

**Figure 3:**
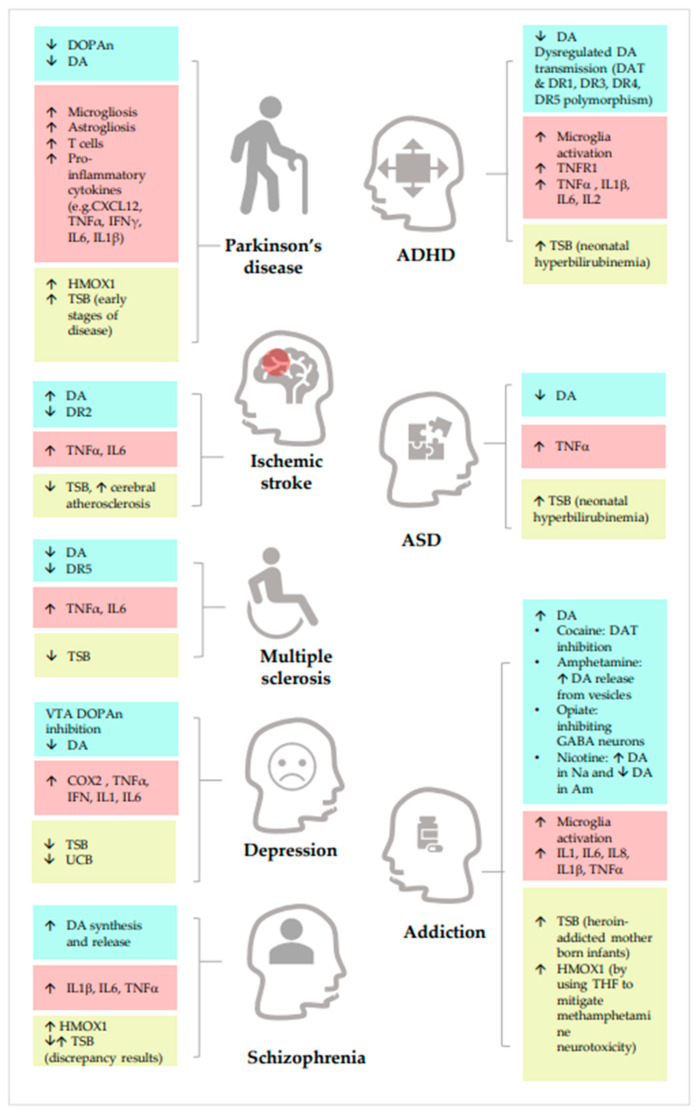
The alteration in dopamine (light blue), inflammation factors (pink), and YPs (yellow) in DA-related diseases. ↑: increase; ↓: decrease; DOPAn: dopaminergic neuron; DA: dopamine; CXL12: C-X-C Motif Chemokine Ligand 12, TNFα: tumor necrosis factor α, IFNγ: interferon γ, IL6: interleukin 6, IL1β: interleukin 1β; HMOX1: heme oxygenase 1; TSB: total serum bilirubin; DR: dopamine receptor: DR5: dopamine receptor 5; COX2: cyclooxygenase 2; IFN: interferon; IL1: interleukin 1, UCB: unconjugated bilirubin, DAT: dopamine transporter; DR1: dopamine receptor 1, DR3: dopamine receptor 3, DR4: dopamine receptor 4, IL2: interleukin 2; GABA: gamma-aminobutyric acid; Na: nucleus accumbens, Am: amygdala; IL8: interleukin 8, THF: of 6,7,4′-trihydroxyflavanone. ADHD: Attention Deficit Hyperactivity Disorder; ASD: autism spectrum disorder.

Extensive clinical and experimental evidence supports the involvement of inflammation in the onset and progression of PD. Microgliosis, astrogliosis, and the CD8+ and CD4+ adaptive immune T cells present in the brain of PD specimens (human and models) can lead to the induction of inducible nitric oxide synthase (NOS) and cyclooxygenase (COX) alongside the release of the pro-inflammatory cytokine C-X-C motif chemokine ligand 12 (CXCL12), TNFα, interferon-γ (IFNγ), IL6 and IL1β [44,79,80,81]. Specifically, TNFα has been suggested to be a relevant player in PD. TNFα elevation has been reported in both postmortem human PD specimens and models. It has been associated with an enhanced DOPAn demise, physical and cognitive decline severity, and correlated with the Hoehn and Yahr score [82,83,84,85,86,87] (Figure 3). Recently, an SNP (single nucleotide polymorphism) in the TNFα promoter gene (-1031C) has been associated with a rare form of early-onset idiopathic PD [88]), and TNFα (G308A) polymorphism has been associated with vascular Parkinson’s complicated with pulmonary infection [89]. Interestingly, in the young onset forms of PD, symptoms frequently develop secondary to infectious diseases, stressing even more the association between the activation of the inflammatory cascade and DA neurodegeneration. Between 5 and 10% of PD cases may have a genetic cause. Again, interestingly, one of the most recent perspectives on the etiology of this disease has suggested that the genes involved in PD may share biochemical mechanisms or signaling pathways with inflammatory pathways [28,90,91,92,93].

Levodopa, DA agonists, MAO (monoamine oxidase) inhibitors, and other non-pharmacologic therapies mainly target a boost in dopamine production or inhibit its catabolism to alleviate the symptoms [28,44,94]. However, a cure is still definitely an unmet clinical need. The administration of antioxidants looks not to affect the course of PD, and the effect of statins (considered to act as anti-oxidants) is inconclusive, suggesting that redox stress might not be the primary responsible for DA reduction. Regarding these anti-inflammatory approaches (Table 1), non-steroidal anti-inflammatory drugs (NSAIDs) have been reported to lower up to 45% of PD disease risk [78]. Ibuprofen reduces up to a 27% PD incidence, possibly acting through the activation of PPARγ [78] and decreasing DA turnover [76]. A similar result has been suggested for indomethacin: a weak agonist of PPAR [95]. Interventions working at the systemic level in decreasing systemic inflammation (see Section 4 for understanding the rationale) have also been considered. Moderate or vigorous physical activity is associated with a 34% reduced risk of PD, possibly by increasing BDNF release and DA synthesis [78] and/or decreasing the systemic inflammatory status [76,96]. Probiotics (lactobacilli, enterococci, bifidobacteria, yeasts, and various mixtures of beneficial bacteria) have been suggested to rebalance a PD-associated change in the microbiota composition, reducing the leaky gut and inflammation [96]. Tobacco (nicotine at a low dose, while at a high dose, it damages the BBB [76]) enhances TH expression and the DA level in PD through PPARα. Still, the nicotine stimulation of the reward system explains Add [27,97]. Caffeine, theophylline, and theobromine increase DR activity in PD, decreasing the risk of developing the disease [76]. Based on the number of anti-inflammatory approaches and the results, even if partial, there is no doubt that PD is an inflammatory disease and that inflammation is a therapeutic target.

YPs have also been shown to participate in PD and inflammation. A comprehensive picture of PD, inflammation, DA, and YPs interplay could be recomposed by putting together the information belonging to different works. TSB is increased in the early stages of PD when it negatively correlates with the severity of symptoms and with less need for L-DOPA administration [98]. TSB has also been associated with the demise of DOPAn in models [99]. Moreover, in the brain parenchyma, the HMOX1 signal has been documented to be increased in specific areas (SNpc, regions of the neocortex with Lewy bodies) and cells (DOPAn, microglia, and astroglia) affected by the disease, and correlated with reduced inflammation, in agreement with the protective, anti-inflammatory action of bilirubin. On the contrary, (a) a TSB decrease even below the level of the controls has accompanied the progression of this disease to the most severe stages; (b) genetic polymorphisms of HMOX1 have also been shown to decrease BV and bilirubin production, correlating with the early onset of PD, stressing once again the importance of YPs and the modulation of inflammation to prevent or delay the pathology [100,101,102,103,104,105]. Based on this evidence, increased TSB, the increased presence of bilirubin degradation products in the urine, and an increased HMOX1 expression level have been suggested as potential markers for PD [35,103]. In models, bilirubin has been documented to induce TH enzyme and to increase DA levels in vivo (by NFκB signaling pathway [106]). In turn, DA up-regulates the HMOX1 expression in vitro (endothelial cells [107]). Interestingly, DA may also inhibit the canonical, non-canonical, and α-syn-mediated activation of the NLRP3 inflammasome in vitro: a mechanism involved in DOPAn loss. Similar to the induction of HMOX1 and bilirubin increase in the early stages of PD, the inhibition of the inflammasome operated by DA might be interpreted as a tentative protective reaction to ongoing damage [108]. Notably, NRF2 is a primary regulator of cellular defense against oxidative stress and inflammation in DOPAn neuroprotection [18] and is one of the major signaling pathways of bilirubin protective action too [30] (Figure 2). On the other hand, the inhibitory effect of bilirubin on several steps of DA biology (tyrosine uptake; cAMP-stimulated DA synthesis, but not basal production; DA reuptake and cellular vesicular storage) has also been reported [109,110,111,112]. Part of these experiments have been conducted using toxic concentrations of bilirubin (70–140 µM). This might explain the contradictory results. To this note, a harmful amount of bilirubin, as in severe neonatal hyperbilirubinemia, has been reported to trigger attention deficit hyperactivity disorder (ADHD), schizophrenia (Sch), and autism spectrum disorder (ASD) in adult life, which are all known conditions in which DA is central, suggesting that a toxic amount of bilirubin could negatively affect one or more DA circuits (see later). It is worth mentioning that, in 2019, Chang et al. investigated the potential of idiopathic hyperbilirubinemia and Parkinson’s disease to be associated with negative (lack of association) results [113]. In the Gunn rat, a spontaneous model of severe neonatal hyperbilirubinemia, DA evaluation in different parts of the brain gave contradictory results. DA concentration in the striatum, pons, medulla, and cerebellum was diminished based on Swenson [114] but was unaltered in the striatum and frontal cortex based on Shoko Miura [115]. This PD, DA, YPs, and inflammation interplay narrative needs experimental confirmation. Up until now, to the best of our knowledge, two of our works have supplied strong enough evidence on the protective role of supplementing tissue with bilirubin, counteracting inflammation. In organotypic brain cultures, models of PD (induced by rotenone) and inflammatory modulation (namely IL6, TNFα, and COX2), together with HMOX1 induction, precedes DA demise [81]; low (non-toxic) bilirubin supplementation fully protecting DOPAn specifically acts on TNFα [116]. Multiple pieces of evidence have supported these findings. NAC, as an anti-oxidant, is ineffective a protecting DOPAn. b) Infliximab (a clinic antibody neutralizing TNFα) administration to PD slices fully inhibits DOPAn demise. c) TNFα administration to healthy cultures induces a dose-dependent DOPAn loss identical to rotenone challenging. The question of whether this applies to other DA-related conditions has to be empirically evaluated. The following paragraph could serve as a background for understanding the potential of YPs and inflammation modulatory approaches in each condition. For completeness, it has to be remembered that the excessive activity of HMOX1 could lead to iron deposition and worsen the damage. DOPAn are not only intrinsically or highly sensitive to stress; however, DA itself might be easily oxidized, amplifying ongoing redox damage [1,42,103,117,118,119,120,121,122,123,124,125,126]. Thus, YPs and DA dysregulation might hypothetically result in a potent damaging couple in neurologic conditions. The data on YPs’ potential in PD strongly suggests that the supplementation of exogenous bilirubin, for example, by nanobubbles, rather than inducing YPs’s enzymes, could be a safer therapeutic approach.

### 3.2. Schizophrenia

Schizophrenia (Sch) is characterized by psychotic symptoms such as hallucinations, delusions, and disorganized speech, with negative symptoms including demotivation, reduced expressiveness, and cognitive deficiencies including impaired executive functioning, memory, and mental processing speed [127]. Positron emission tomography (PET) has shown an increase in DA synthesis and release in the dorsal striatum in individuals with Sch [128], supporting the role of DA in Sch as a long-prevailing hypothesis. This concept is further supported by a gene and transcriptome wide-association study of the postmortem brain, in which the gene encoding the dopamine DR2 was reported as a risk factor [129]. Psychotic symptoms tend to diminish in Sch patients who develop PD [130]. On the contrary, dopaminergic therapy induces psychosis in PD, and anti-psychotic therapy usually gives Parkinsonism as a side effect, highlighting the positive connection of DA with Sch (more DA, more Sch features) [131,132,133]. For this reason, the coexistence of both diseases challenges the treatment balance [134,135] (Figure 3).

The involvement of neuro-inflammation in Sch has been a topic of discussion. Epidemiological studies suggest that Sch (as well as ASD and ADHD) are developmental conditions on which neonatal stress can alter the physiologic synapse formation or neurotransmission development, with neurologic manifestations appearing in adult life. In line with this, pre-natal inflammation has been associated with an increased risk of Sch [136,137], with detrimental effects mediated by IL1β, IL6, and TNFα [138,139,140]. Moreover, a pro-inflammatory status led by the gut was observed in patients with Sch, and the abundance of specific bacterial genera, Succinivibrio and Corynebacterium, has become significantly associated with the symptom severity of these diseases [141]. Concerning specifically TNFα, its serum level was found to be significantly higher before anti-psychotics treatment in first-episode drug-naïve Sch patients than in chronic patients and healthy controls, suggesting that an increase in TNFα was probably an early mechanism in the development of this disease [142]. This was supported by Lv et al., who reported a decrease in TNFα serum levels in chronic schizophrenia on long-term antipsychotics compared to healthy individuals [143] (Figure 2). Concerning the anti-inflammatory interventions in Sch, minocycline improved symptoms but not cognitive functions and aspirin has given contradictory results [144]. Statins (cholesterol-lowering agents, lowering also IL1β, IL6, TNFα, and CRP levels) may alleviate anxiety-like behavior in models of Sch by up-regulating the NMDA receptors, with improvements also reported in clinical trials, while in a study based on two subjects IFNγ reduced the negative symptoms [144]. Omega-3 fatty acids (docosahexaenoic acid—DHA, eicosapentaenoic acid—EPA; anti-oxidant, suppressing NFkβ and reducing the levels of IL1, IL6, and TNFα [145]) have provided contradictory results in Sch [144,146]. Possibly relevant to this review, celecoxib, as a selective blocker of COX2, looks to improve behavioral impairments in rodent models by reducing TNFα (Table 1). Its effects on DA levels have not been directly assessed, and its improvement is now referred to as the prevention of parvalbumin (PV) in calcium-binding GABAergic neuron loss [144]. Of relevance to this review, these neurons are one of the most hypothesized targets of bilirubin neurotoxicity in severe neonatal hyperbilirubinemia. This condition has also been suggested as a potential cause of certain diseases (see later).

A discrepancy exists in the findings on TSB levels in Sch. Some examples in the literature report a decrease in TSB [147], while others identify increased TSB levels in subjects without suspicion of genetic reasons for increased bilirubin in the blood [148]. A positive correlation (higher TSB, higher Sch incidence) also looked to be present in Gilbert’s subjects, with a uridine glucuronidase (UGT1a1) defect that led to mild hyperbilirubinemia [149]. The genetic polymorphisms of the UGT1a1 enzyme in the Gilbert population could contribute even directly to DA concentration in the brain, with DA a substrate for the UGT1a mediated conjugation and dopamine glucuronide found in rat and mouse brain samples [150]. Even though it is still arguable, neonatal hyperbilirubinemia has been linked to schizophrenia in adult life, which is in agreement with the developmental hypotheses of Sch [151]. In agreement, the Gunn rat, the animal model of neonatal hyperbilirubinemia, has been used as a disease model as it presents Sch-like behavior [152,153]. In a recent publication, we reported that bilirubin mainly affected glutamate circuits in the brain, further supporting the potential link between higher bilirubin levels early after birth and the developmental hypothesis of schizophrenia [151]. The overexpression of HMOX1 in mice astrocytes resulted in DA augmentation and DR1 reduction and also has been linked with Sch-like features [154].

In summary, while increases in DA deficiency and TNFα cause motor PD, with the beneficial action of bilirubin, the excess DA with increased TNFα has recently been linked to Sch-related psychosis (Figure 2 and Figure 3).

### 3.3. Attention Deficit Hyperactivity Disorder

With a prevalence of over 5%, attention deficit hyperactivity disorder (ADHD) is one of the most frequent disorders in child and adolescent psychiatry [155]. ADHD’s clinical manifestations include a lack of attention, impulsivity, excessive motor activity, and hyperactivity [156]. Environmental etiological factors, including exposure to environmental toxins like organophosphate pesticides, polychlorinated biphenyls, and zinc, as well as prenatal and postnatal factors like maternal smoking and alcohol use, low birth weight, and premature birth, have all been linked to an increased risk for ADHD in past years [157]. The activation of TLR4 appears to be central [158]. A gene-by-environment interaction has been observed between the DAT1 (dopamine transporter 1) genotype and prenatal smoke exposure regarding hyperactivity—impulsivity [159,160]. DAT and dopamine receptor (DR1, DR2, DR4, and DR5) polymorphisms have been associated with an increase in ADHD [156,161,162,163,164]. In general, DA reduction and dysregulated DA neurotransmission, particularly between the prefrontal and striate areas, are the molecular fingerprint of ADHD [165,166].

The potential involvement of neuroinflammation in ADHD has been discussed, and additional studies are needed for a more precise conclusion. Nevertheless, data on inflammation and ADHD are available. Neuroinflammation has been proposed as a risk factor for ADHD, as inflammation-related diseases such as atopic immune disorder, asthma, and rheumatoid arthritis, in addition to the perinatal conditions listed before, have been associated with the neuropsychiatric condition [167,168,169,170,171]. Particular emphasis on maternal inflammation in obesity and offspring neurodevelopmental disorders (ADHD and autism spectrum disorder) has been taken by Velda et al., who reported the activation of TLR, MAPK, NFkβ, and microglia, together with the epigenetic modulation of the brain environment which could have a critical role [158] (Figure 2 and Figure 3). In agreement, a higher level of TNF-R1 (the receptor of TNFα) was found to be associated with ADHD [172], and a positive correlation between the TNFα serum level and hyperactivity-impulsivity was found in ADHD subjects [173]. Anand also explained ADHD as a pro-inflammatory condition (microgliosis, IL1β, IL2, IL6, and TNFα), potentially affecting the development and/or functioning of the CNS, and finally reducing DA availability, in which perinatal infections are a relevant co-cause. The reduction in the neurotransmitter and the symptomatology could be mimicked in models by the injection of cytokines (IL1β, IL2, and IL6) [168]. ADHD is also known as a neurodevelopmental condition. For example, the FOXP2 (forkhead box protein P2) gene, which controls DA and neurodevelopment in brain regions related to ADHD, was discovered to be strongly associated with ADHD in GWAS studies [174,175]. A reduction in the volume of the nucleus accumbens, amygdala, caudate, hippocampus, and putamen has been described in patients with ADHD [176].

Of relevance to this review, ADHD has been reported to be possibly induced by a post-natal exposition to the toxic bilirubin level. Chang-Ching Wei et al. reported a 2.48-fold increase in ADHD in the cohort of neonates presenting TSB levels requiring phototherapy and with longer admission days vs. the non-jaundiced cohort. The risk of neurologic sequelae was more remarkable for male, preterm, and low-birth-weight infants with neonatal jaundice [177]. A 30-year prospective study also unraveled that hyperbilirubinemia in early life could recreate a picture resembling the impairment of the frontostriatal network and the symptoms of the ADHD spectrum [178]. Indeed, bilirubin has been shown to have a dose-dependent inhibitory impact on the absorption of tyrosine: a precursor to dopamine [109]. Bilirubin has also been found to inhibit cAMP-stimulated dopamine synthesis and decrease its absorption and vesicular storage [110,111,112] (Figure 2).

Moreover, bilirubin-induced neurotoxicity is known to occur via the activation of microglia, astrogliosis, and the release of pro-inflammatory mediators, including TNFα, leading to developmental impairment, especially (but not limited to) basal ganglia and cerebellum [177,179], and as discussed in Sch, through the transcriptomic imprinting of genes that are involved mainly on glutamate neuro-transmission [151]. It suggests that at least high amounts of bilirubin may be dangerous in the context of ADHD. The effect of low (protective) doses of bilirubin (Gilbert-like of physiologic jaundice) or the modulation of YPs in the brain on ADHD is unknown. To complete the section, based on results in animal models, caffeine has also been suggested as an optional approach in this condition, acting on several mechanisms, including DA paired-adenosine receptors A_2AR,_ DAT level, and DA uptake at synapses. Of relevance for this review is among the reported targets of caffeine in ADHD, PKA, and PI3K, which are shared with bilirubin [180].

### 3.4. Autism Spectrum Disorder

During the past few decades, there have been indications of an increased prevalence of the neurodevelopmental disorder autism spectrum disorder (ASD) [181]. ASD has been linked to changes in dopamine signaling. Particularly changes in the mesocorticolimbic dopaminergic signaling system have been observed in autistic individuals, including decreased DA release in the prefrontal cortex and a decreased neuronal response in the nucleus accumbens [182,183]. Several studies have reported that TNFα concentrations also increase in the blood, cerebrospinal fluid, or post-mortem brain samples of ASD subjects [184,185,186,187]. Moreover, children with autism display an elevated blood TNFα concentration correlating with symptom severity [188] (Figure 3). As previously mentioned, in agreement with the concept of the developmental disorder, an increased risk of ASD is present in infants exposed to maternal immune activation during pregnancy [158].

Also, for this condition, a negative correlation with TSB has been reported [179]. In a meta-analysis study (13 papers) on neonatal jaundice, the TSB level in the blood was associated with ASD in term infants but not in pre-terms, despite their moderate (10 mg/dL) TSB level [189]. Notably, similarly to ADHD, ASD is considered a neurodevelopmental disorder. The suggested adverse effect of bilirubin in DA, glutamate, and development biology could also be applied to ASD.

### 3.5. Depression

Depression (Dp, in figures) is one of the most serious mental disorders and is a major factor in the rising suicide rate in the 21st century, as a widespread mental disorder that jeopardizes the global population’s physical and psychological health [190]. Recent research has revealed that the pathophysiology of depression might be caused by disturbances in DA regulatory networks, specifically the DOPAn populations implicated in motivation and reward. Some antidepressant drugs and brain stimulation therapies can impact the intricate DA system, with the selective inhibition of VTA DOPAn demonstrated to induce depression-like behavior in rodents [191].

According to clinical studies, peripheral inflammatory cytokines and their soluble receptors have been reported to be increased in the blood and cerebral fluid of individuals with major depression [192]. Cytokines may alter a range of dopamine neurotransmission processes, impairing vesicular DA packing and their release or increasing reuptake, which could interact to varying degrees to diminish basal ganglia dopamine neurotransmission [47]. Again, systemic inflammation (such as in metabolic syndrome) has been suggested to alter the brain DA circuit involved in depression and anhedonia, with the CNS modulation of IFN, IL1, IL6, TNFα, and COX2 levels, as a supposed critical role for TNFα and IL6 [193,194,195]. The results in models support this, where blocking two cytokines looks pivotal in reversing depressive symptoms [193]. Specifically, TNFα is known to damage the BBB, contributing to the development of the condition [196] (Figure 3). The pathophysiology of depression is thought to be mediated by TNFα signaling through the binding to its receptor TNFR1 [197]. In agreement, Infliximab, as a TNFα neutralizing antibody, can alleviate depressive symptoms in patients with treatment-resistant depression and elevated inflammatory markers [198]. Similarly, celecoxib, as a COX2 inhibitor, exhibited a satisfactory therapeutic effect in patients with this disease [199]. Aspirin improves anti-depressants’ effect [195], possibly by MAPK modulation [200]. Minocycline application to depression has also resulted in inconclusive results [47,193], similar to statins and omega-3 fatty acids [193,195]. Combining ibuprofen and an anti-depressant gives disputed results [78,195] (Table 1).

Several studies have explored the association of bilirubin with depression. TSB has been reported to be significantly lower in major depressive disorder subjects and has been suggested as a result of bilirubin overconsumption to be an antioxidant [201]. Indeed, a low nocturnal bilirubin level is associated with winter seasonal depression [202].

Again, the picture of a decreased DA and increased inflammation, including TNFα, and reduced bilirubin, looks supportive of the possible beneficial anti-inflammatory action of pigment supply in depression.

### 3.6. Addiction

Addiction (Add) is regularly identified with the habitual nonmedical self-administration of drugs [203]. DA has typically been linked to the reinforcing effects of addictive substances and could play a significant role in initiating the neurobiological alterations connected to addiction [165]. Different mechanisms of action on DA biology have been identified; however, in general, cocaine, amphetamines, opiates, alcohol, nicotine, and caffeine can elevate the extracellular neurotransmitter level in the Na core and Na shell in the ventral striatum region, which receives dopaminergic innervation from the ventral tegmental area (mesocorticolimbic DA system [204]), inducing a reinforcement effect [72,205], which is the basis of habit-forming [204]. To stress once more the link between systemic and CNS disease (see later), the same picture was documented in obese people with metabolic syndrome [206,207]. This supports the potential of shared neurologic mechanisms on the basis of addiction to drugs and “addiction” to palatable foods [208]. Specifically on the effects of abuse substances on DA biology, cocaine increases extracellular dopamine levels by preventing DAT from reabsorbing dopamine [203]. Amphetamines increase extracellular levels by releasing DA from vesicles [203]. Opiates activate the DA system by inhibiting GABA neurons which normally hold the dopamine neurons under inhibitory control [203]. Alcohol and cocaine increase DA release [76,209]. Similarly, nicotine induces Add by increasing the release of DA in Na while also promoting anxiety by inhibiting DA neurotransmission in Am [210].

It is believed that neuro-inflammation has a role in the neuronal changes that result from the long-term misuse of drug exposure [200], with morphological and functional alterations in the microglia and astrocytes characterizing the neuro-immune response to drugs in Add [211]. Microglia activation triggers cell migration to the site of injury, phagocytosis, and the production of pro-inflammatory mediators (IL1, IL6, and TNFα), as well as ROS and NOS that harm neurons [212,213,214]. Based on the translocator protein (TSPO—a marker of activated glia) neuro-imaging in methamphetamine users, the DA alterations are mediated by an increased inflammatory status (with IL6, TNFα release, and NOS and ROS generation), possibly through the increase in PPARγ activity and protein levels, which results in increased release of IL1β, IL6, and TNFα in the Na [212,213,214,215] (Figure 2 and Figure 3). TNFα inhibits an increase in extracellular DA levels caused by methamphetamine while also activating plasmalemma and vesicular DA transporters, which could help prevent the drug dependence and neurotoxicity induced by methamphetamine [216]. Conversely, a pro-inflammatory CNS environment, with an increase in TNFα, could alleviate methamphetamine addiction [217]. Cannabis is largely considered therapeutic based on its anti-inflammatory effect [218], but increases in IL1β, IL6, IL8, and TNFα have also been documented [219]. These discrepancies depend on the age at initiation of use [200]. For the potential benefits of other molecules, see Table 1 [25,27,97,220,221] (Table 1).

Very few data exist on YPs’ effects in Add. Interestingly, 6,7,4′-trihydroxyflavanone (THF), a flavone present in Leguminosae, might mitigate methamphetamine neurotoxicity by enhancing the NRF2 and PI3K signaling pathways, inducing HMOX1 expression and reducing oxidative stress and apoptosis in an in vitro neuronal model [222].

### 3.7. Ischemic Stroke

An ischemic stroke (IS) is characterized by a reduced brain blood and oxygen supply [223].

After an IS, a large DA release might be responsible for the neuronal damage brought on by ischemia through the activation of DR2 [224], followed by a smaller DA release for up to 3 days [225]. Subsequently, the genes that encode DR are down-regulated for at least one week [226]. One and two weeks after middle cerebral artery blockage, the striatum consistently exhibits decreased DR2 availability [227]. These findings can relate stroke to DA impairment, which could explain why people with a stroke have reduced implicit learning [228]. Moreover, the inhibition of dopamine DR1 and DR2 impaired motor skill recovery in an ischemic stroke model, implying the role of endogenous dopamine transmission in this rehabilitation activity (Figure 3).

Redox stress has been primarily implicated in DA-mediated neuronal sufferance. DR2 is a member of G protein-coupled receptors, which regulate cellular redox homeostasis [229]. Therefore, DR2 receptor agonists have neuroprotective potential by counteracting redox imbalance and mediating the anti-apoptotic process [230]. Indeed, the administration of DA DR2 agonists prevents neuron death by decreasing mitochondrial ROS production [231]. Little information is available on inflammation in stroke and DA. In this respect, it has been reported that glutathione-mediated intrastriatal DA production could reduce the cerebral infarction area and decrease inflammatory cytokines levels, including IL6 and TNFα [232], suggesting the damage-enhancing role for inflammation in the DA consequences of stroke. Parikh et al. recently reviewed the epidemiologic evidence of inflammation, autoimmunity, and infections on IS, reporting a possible correlation and partial result using anti-inflammatory approaches [233] (Figure 3 and Table 1).

The relationship between bilirubin and IS has been reported in some studies. Based on a Mendelian Randomization study, an inverse causal association between TSB and the risk of IS was shown [234]. Supporting these previous findings, a study on asymptomatic intracranial atherosclerosis as one of the IS risks found that participants with TSB lower than 12.30 uMol/L (0.72 mg/dL, with a normal range usually of 0.4 to 1.0 mg/dL) had an increased risk of developing cerebral atherosclerosis compared to those in the high (more than 12.3 uMol/L = 0.72 mg/dL) concentration group [235]. Of notice, only recently, low levels of TSB (in the lower normal range or below) have been proposed as a marker of risk for multiple neurologic conditions [35,80,236]. Of importance for this disease, the well-known anti-oxidant properties of bilirubin could be helpful in IS [237,238,239]. Nevertheless, considering the rise in DA at the site of the lesion, the hyperactivation of HMOX1 has to be avoided. As anticipated (see above), HMOX1 activation led to a rise in extracellular iron concentration, a potentially explosive mix. Again, the delivery of bilirubin but not the stimulation of YPs might be a reasonable approach.

### 3.8. Multiple Sclerosis

Multiple sclerosis (MS) is the most prevalent demyelinating condition affecting the central nervous system [240], in which DA is one of the most studied neurotransmitters in the modulation of neuro–immune interaction. Carandini et al. demonstrated that axonal damage in people with relapsing-remitting MS affects DA and norepinephrine neurotransmission [241]. Moreover, DA is also dysregulated in the peripheral immune system in MS [242]. In individuals with chronic progressive MS or relapsing-remitting MS, DA synthesis is decreased in activated lymphocytes [8]. In the peripheral blood mononuclear cells (T cells, B cells, and NK cells) of untreated relapsing-remitting MS patients, DR5 protein and mRNA levels are decreased [243].

Inflammatory cytokines seem to affect fatigue, as the neuropsychological symptoms of MS. Relapsing-remitting MS patients with fatigue were found to have greater plasma levels of IL6 and TNFα than the control group (relapsing-remitting MS patients without fatigue), and both cytokines concentrations were associated with fatigue severity [244]. A higher level of TNFα was also found in the CSF of MS patients compared to the controls [245]. IFNβ is an immunomodulatory drug for relapsing-remitting MS, decreasing DA synthesis [246] (Figure 3).

In clinical settings, a decrease in TSB is frequently noted in MS patients [80]. As discussed in IS, a lower TSB has recently been hypothesized as a risk factor for neurologic diseases [30,35], and the potential biological explanations are described (see above). Studies on the animal model of MS, autoimmune encephalomyelitis (EAE), have described the protective function of bilirubin and YPs in this condition. Those studies demonstrated that the induction of the bilirubin-producing enzymes, HMOX1 and BLVR, reduced the symptoms of EAE, which acted on both the inflammatory and oxidative stress injury, protecting BBB integrity and reducing the invasion of inflammatory cells into the spinal cord [247,248,249]. Lately, more vital evidence of the anti-inflammatory effect of bilirubin was obtained by Kim et al., who injected bilirubin nanoparticles with the resulting mitigation of EAE progression by downregulating dendritic cells and Th17 production [250]. In conclusion, MS studies provide evidence that a low TSB may be a marker for the risk of disease progression, and the anti-inflammatory action of YPs might provide protection.

### 3.9. Late Neurologic Sequel of Neontal Hyperbilirubinemia

Because in this review we have discussed the possible positive (restoring, alleviating, protective) interplay of YPs with inflammation and DA, it is mandatory to mention that severe neonatal hyperbilirubinemia has been supposed to correlate with DA neuropsychiatric syndromes, namely ADHD, Sch, and ASD. Despite this being a neglected area of research in the bilirubin field, some solid epidemiologic studies may be found [177,178,189,251]. For all these conditions, it has to be pointed out that, in severe neonatal hyperbilirubinemia, the beneficial effect of bilirubin turns upside down, with bilirubin becoming highly pro-inflammatory and interfering with brain development [153]. We have reported on the permanent transcriptomic alteration of genes involved with the aforementioned neurologic diseases [151], supporting the potential of a toxic amount of bilirubin in inducing neuropsychiatric conditions. Further studies are needed, especially if bilirubin should be considered as a therapeutic option.

### 3.10. Cerebral Palsy

Cerebral palsy (CP) is defined as a progressive injury to the developing CNS in children, leading to neurological and musculoskeletal abnormalities with severe motor disorders, including spasticity, dystonia, and immobility [252]. Prematurity, infections, and inflammation (TNFα/β, IL6, IL10) have been documented [253,254], and an increased TNFα level suggested a negative correlation with rehabilitation outcome [255].

The mesocortical DA circuit controls learned motor skills in humans [256] and models on which CREB/PKA signaling pathways have been suggested to be central [257]. The DA involvement in CP was supported by a study that assessed the association between successful rehabilitation (involving motor-learning abilities) and defined genetic polymorphisms on genes involved in DA biology (COMT—rs4680, DAT—rs28363170, DRD1—rs4532, DRD2—rs1800497, DRD3—rs6280) on 33 patients with spastic unilateral CP [258]. The association between DA genetic variants and motor skills in CP infants was corroborated in 498 extremely low-birthweight infants [259]. Indeed, DA is a treatment in children with dystonia and CP [252].

CP is also a known feature of severe neonatal hyperbilirubinemia, with bilirubin reported to damage the basal ganglia and cerebellum [260,261]. Notably, the cerebellum is also a central region in controlling body movements. Currently, there are no studies that assess the effect of severe neonatal hyperbilirubinemia and DA biology in CP. Nevertheless, as discussed in Section 3.1, the DA level was found to decrease in the striatum, pons, medulla, and cerebellum of the Gunn rat [114]. Together with the known pro-inflammatory effect of sustained TSB, and the shared target signaling pathways, this suggests the involvement of high (toxic) TSB, inflammation, and DA dysregulation in the development of neonatal CP.

### 3.11. Other Conditions Linked to DA Biology

For the other conditions due to alterations in the DA system, such as the restless leg syndrome, minimal hepatic encephalopathy, pain potentially inducing anhedonia-like behavior, and the emotional and cognitive deficits involving the mesocortical dopaminergic system, no data support a discussion on DA, inflammation or the interplay of YPs.

**Table 1:** Interventions with beneficial effects on DA-related diseases.

Types	Drugs/Compounds	DA-Related Disease	Ref.
**Anti-inflammatory drugs**	NSAIDs	PD	[78]
	Ibuprofen	PD, Dp	[78,195]
	Indomethacin	PD	[95]
	Celecoxib	Sch	[144]
	Aspirin	Dp, Add	[95]
	Minocycline	Sch	[144]
	Statins	Sch	[144]
	IFNβ	MS	[246]
	IFNγ	Sch	[144]
	TNFα antagonists	Dp	[198]
**Physical activity**	-	PD	[76,78]
**Nutritional interventions**	Omega-3 fatty acids	Sch, Dp	[174,193,195]
	Oleoylethanolamide (OEA) and palmitoylethanolamide (PEA)	Add	[25,27,220]
	Probiotics	PD	[96]
**Others**	Tobacco	PD	[76]
	Caffeine	PD, ADHD	[76,180]
	Theophylline	PD	[76]
	Theobromine	PD	[76]
**YPs modulation**		PD, Dp, MS	[44,80,116]

NSAIDs: non-steroidal anti-inflammatory drugs; IFN: interferon; TNF: tumor necrosis factor; YPs: yellow players; PD: Parkinson’s disease; Dp: depression; Sch: schizophrenia; Add: addiction; MS: multiple sclerosis; ADHD: Attention Deficit Hyperactivity Disorder.

## 4. DA, Systemic Inflammation and the Brain

A recent vision on neurologic conditions has suggested that the brain is not as isolated as it has been said and that systemic stimuli may either affect or even trigger neurologic diseases.

DRs are expressed by T-cells, dendritic cells, B cells, osteoclasts, neutrophils, eosinophils, monocyte, and macrophages [11,22], which link together DA and systemic inflammation (infections, sepsis, colitis, rheumatoid arthritis, tumors, liver injury, metabolic syndrome, inflammatory bowel diseases, Chron disease, diabetes, etc.) to neurologic conditions (Dp, PD, ASD, etc.) [11,14]. Moreover, DA is also synthetized by the mesenteric organs (approximately 45% of all the DA body production), the sympathetic nerves, the gut microbiota (with *Prevotella, Bacteroides*, Lactobacillus, Bifidobacterium, Clostridium, Enterococcus, and Ruminococcus that may modulate DA availability, DA then transported to the brain thanks to the vagus nerve [262,263]), the adrenal medulla, and the kidney [10,264]. Notably, Klingelhoefer and Reichmann even suggested that the pre-motor symptoms (olfactory impairment and constipation) could be explained by the origin of the disease in the enteric nervous system (ENS) before propagating via the parasympathetic trans-synaptic cell-to-cell of alpha-synuclein through the nervus vagus, playing a Troyan horse role [21]. Because the gut is a route for the environment to the body, this may strongly support the potential of environmental substances as triggers of neurologic diseases.

The connection between systemic and brain status in diseases is also supported by metabolic syndrome data. Metabolic syndrome is a growing world problem, especially among young people. In the metabolic syndrome, a diet rich in calories and fat and poor in fibers; microbiota dysbiosis; and low physical activity induce a low pro-inflammatory systemic status, increasing the risk of developing diabetes, cardiovascular diseases (CVD), and CVD-related problems (stroke, ischemia, etc.). Similarly, dietary constituents and a sedentary lifestyle have repeatedly been shown to influence the plasma levels of DA [264,265], strengthening the link between lifestyle, DA, inflammation, the gut, and the brain. Special emphasis on obesity and maternal inflammation concerning offspring neurodevelopmental disorders has been taken by Velda et al. [158]. A link between adiposity and depression has also been suggested and motivated again through an increased systemic inflammatory situation and the increased risk of coronary heart disease [266,267].

While the potential interaction between DA and YPs in the CNS has been discussed above, it is worth mentioning that bilirubin is known to act at the systemic level [30,31,35]. Epidemiologic data (on more than 100,000 subjects belonging to different areas of the world) support bilirubin as an important physiologic modulator of chronic inflammation in metabolic syndrome and diabetes. Gilbert subjects are usually lean and resistant to metabolic syndrome and diabetes type 2. BLVR induction and bilirubin increase have been reported to reduce hepatic steatosis [268,269], possibly through bilirubin binding to PPAR, as a central signaling pathway in obesity, which induces the fat-burning genes Cpt1, Ucp1, and Adrb3 (carnitine palmitoyltransferase 1A, uncoupling protein 1, adrenoceptor beta 3) [35,270]. Also reported is reduced inflammation in the adipose samples, pancreas, and liver, with decreased TNFα and IL1β [271]. The induction of HMOX1 with even a marginal elevation in bilirubin is protective against diabetes type 2 and vascular complications, including retinopathy, nephropathy, and CVD [272], increasing insulin sensitivity, IRS2 expression and regulating its signaling [268,269,273].

## 5. Future Perspective

The inflammation, dysfunctions/diseases of DA biology, and YPs share multiple molecular mechanisms and signaling pathways, both systemically and at the CNS level. Due to its recognized potential for heath, the modulation of YPs is a hot research topic.

YPs, and especially HMOX1, may be modulated, giving the possibility to increase bilirubin levels in the blood [36,274,275,276] and offering a potential clinic application for DA-related pathologies.

The BBB is a limitation to the pharmacologic modulation of YPs in the brain; however, new molecules with better CNS bioavailability can be developed. However, HMOX1 hyperactivation in the brain might be deleterious due to iron deposition with the worsening of the disease.

Bilirubin is known to cross the BBB by passive diffusion from the systemic circulation [277]; however, controlling the level of this pigment that reaches the parenchyma after its systemic increase is not easy, if not impossible. Nevertheless, the documented anti-inflammatory benefits of bilirubin have attracted interest in nanomedicine, and bilirubin-loaded nano-delivery strategies are under investigation with promising results in other contexts (tumors, metabolic syndrome, etc.) [37,278,279,280,281,282,283]. A multidisciplinary (material science, physics, biology, medicine, etc.) approach is essential.

## 6. Conclusions

While in certain neurologic and neuropsychiatric conditions, the connections between DA, YPs, and inflammation are still scarce and need additional studies, and in Sch, bilirubin appears dangerous irrespective of its level, in the larger part of the CNS diseases here reported as well as on their systemic inflammatory component, bilirubin seems to be an endogenous candidate biomolecule for improving or preventing DA related neurologic dysfunctions, supporting the potential for its therapeutic usage.

## Data Availability

All data are contained within the article.
